# Giant Fat-Predominant Uterine Lipoleiomyoma Mimicking a Pure Lipoma: A Multimodality Imaging Case Report

**DOI:** 10.7759/cureus.108656

**Published:** 2026-05-11

**Authors:** Vinutha Honneshaiah, Prajwal M P, Kotresh N S

**Affiliations:** 1 Radiology, Sri Siddhartha Academy of Higher Education, Tumkur, IND

**Keywords:** fat-containing lesion, lipoleiomyoma, mri, transvaginal ultrasonography, uterine lipoma

## Abstract

Uterine lipoleiomyoma is a rare benign lipomatous variant of leiomyoma composed of mature adipose tissue and smooth muscle fibers. Fat-predominant variants are uncommon and may closely resemble pure uterine lipoma on imaging. We report the case of a 44-year-old woman presenting with progressive lower abdominal fullness. Ultrasonography, computed tomography (CT), and magnetic resonance imaging (MRI) demonstrated a large intramural fat-containing lesion arising from the uterine fundus with subtle internal soft tissue strands and no suspicious malignant features. Multiple smaller intramural fibroids were present peripherally. Based on imaging characteristics, a diagnosis of fat-predominant lipoleiomyoma was favored. Given the large size of the lesion and progressive abdominal fullness, the patient underwent a hysterectomy, and histopathological examination confirmed the diagnosis. This case highlights the characteristic imaging findings of uterine lipoleiomyoma and emphasizes the importance of recognizing benign fat-containing uterine lesions to improve diagnostic confidence and avoid unnecessary intervention.

## Introduction

Lipoleiomyoma is a rare benign uterine tumor composed of variable proportions of smooth muscle cells and mature adipocytes and is considered a lipomatous variant of leiomyoma [1,2]. It most commonly occurs in peri- and postmenopausal women, with reported incidence ranging from 0.03% to 0.2% of uterine tumors [1,2]. Fat-predominant variants are particularly uncommon and may mimic other fat-containing uterine lesions such as pure lipoma on imaging [3,4]. These lesions are often incidental but may become symptomatic when large because of mass effect. Imaging, particularly computed tomography (CT) and magnetic resonance imaging (MRI), plays an important role in tissue characterization and preoperative diagnosis [2,4]. We present a case of a giant fat-predominant uterine lipoleiomyoma demonstrating characteristic multimodality imaging findings that closely resembled a pure lipoma.

## Case presentation

A 44-year-old woman presented with a six-month history of gradually progressive lower abdominal fullness. She had no significant past medical, surgical, or gynecological history, including prior pelvic surgery or endocrine/metabolic disorders. Menstrual cycles were regular, and there were no complaints of pain, abnormal bleeding, urinary symptoms, bowel complaints, dyspareunia, or pressure-related symptoms. On clinical examination, a non-tender abdominopelvic mass arising from the pelvis and extending up to the umbilical region was palpable, suggestive of uterine enlargement.

**Figure 1:**
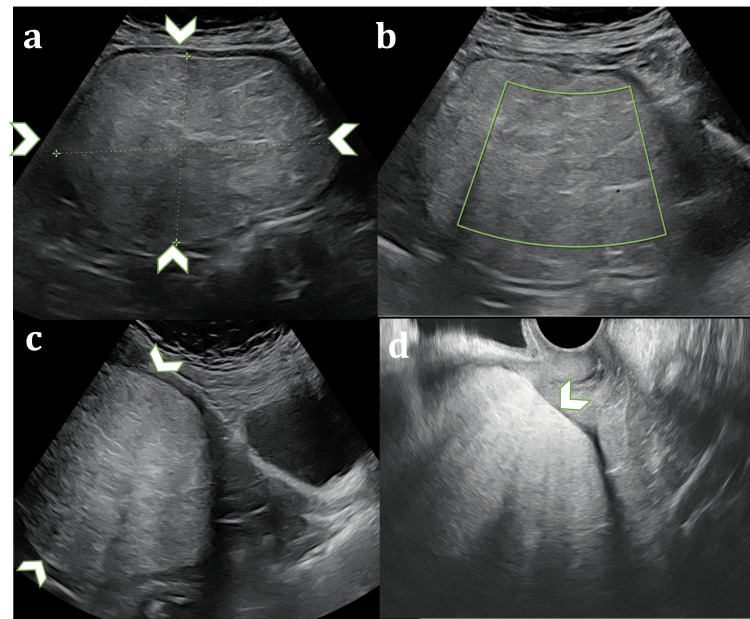
Ultrasonographic features of uterine lipoleiomyoma Transabdominal (a-c) and transvaginal (d) ultrasonography demonstrating a large, well-defined, near-homogeneous hyperechoic lesion (white arrowheads) arising from the uterine fundus, with no significant internal vascularity on color/power Doppler interrogation (b).

Initial transabdominal ultrasonography demonstrated a large, well-circumscribed hyperechoic pelvic lesion, following which transvaginal ultrasonography was performed for better assessment of lesion origin and internal architecture. The lesion measured approximately 13 × 10 cm, arose from the uterine fundus, and was predominantly intramural with a thin surrounding rim of myometrium. No significant internal vascularity was noted on color or power Doppler interrogation (Figure 1).

Given the unusually large size of the lesion and the need for further tissue characterization to exclude other fat-containing pelvic masses, additional cross-sectional imaging with CT and MRI was performed.

**Figure 2:**
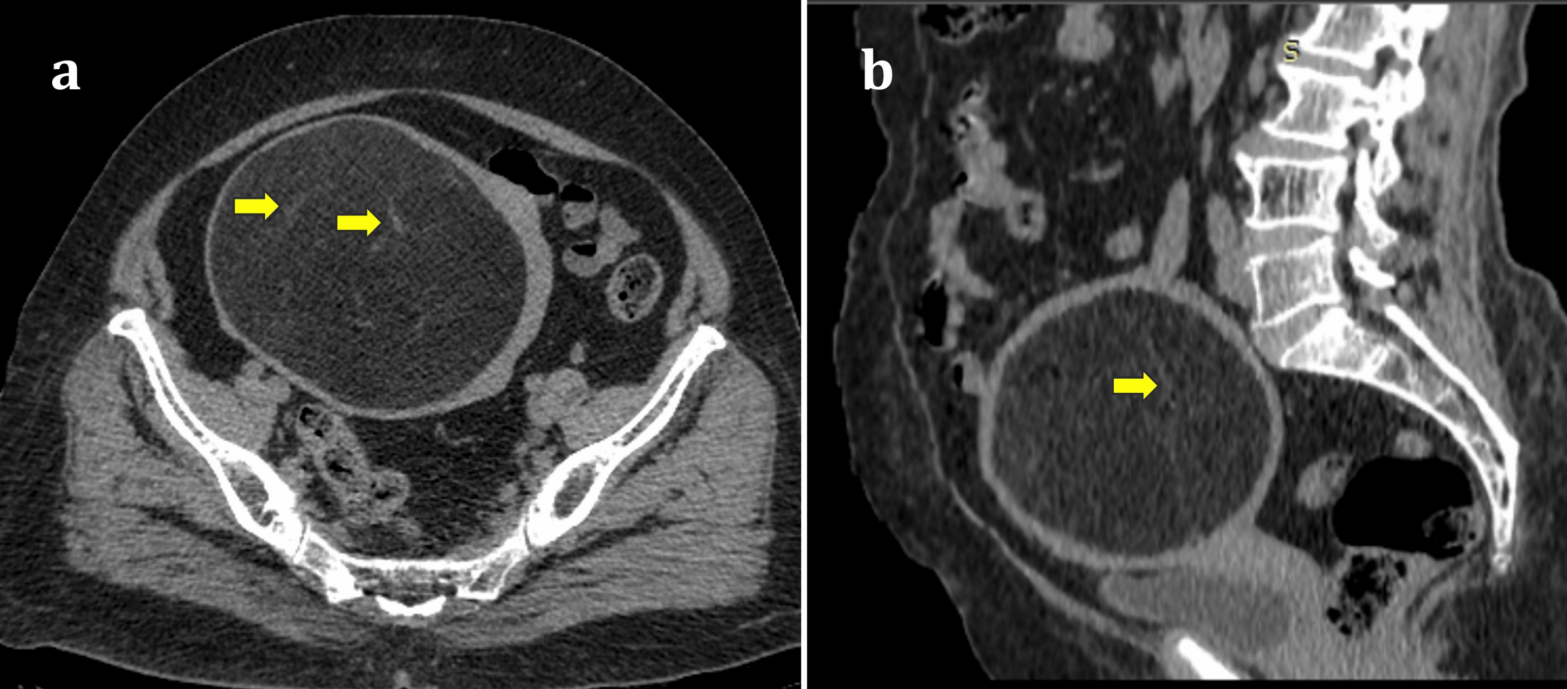
Plain CT features of uterine lipoleiomyoma Axial (a) and sagittal (b) non-contrast CT images of the pelvis demonstrating a large, well-circumscribed hypodense lesion arising from the uterus, showing near-homogeneous fatty attenuation with a few subtle internal soft tissue strands (arrows). CT: computed tomography

Non-contrast CT (Figure 2) of the pelvis revealed a sharply demarcated predominantly intramural uterine mass showing near-homogeneous fat attenuation with a few thin internal soft tissue strands. The lesion demonstrated smooth margins with preserved surrounding fat planes and no evidence of calcification, necrosis, or adjacent invasion.

**Figure 3:**
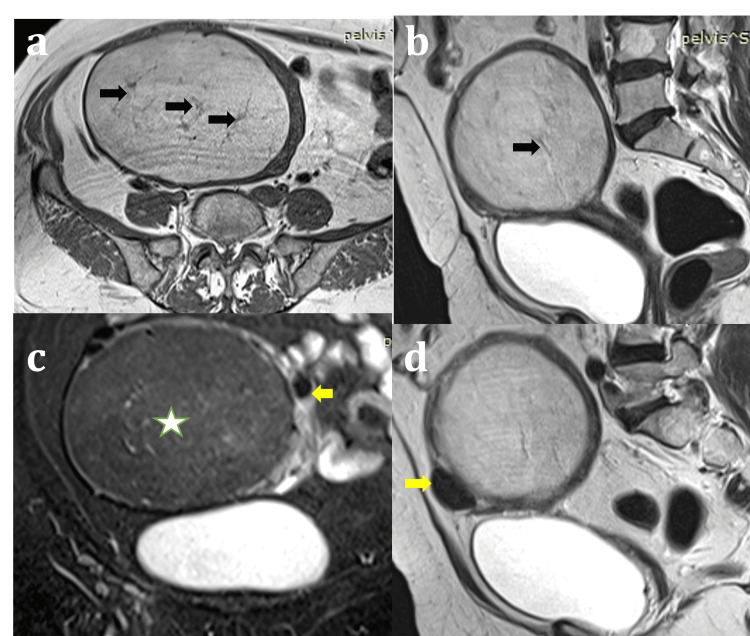
MRI features of uterine lipoleiomyoma Axial T1-weighted (a), sagittal T2-weighted (b), and coronal STIR (c) MR images demonstrating a homogenously T1- and T2-hyperintense lesion arising from the uterine fundus, containing a few internal soft tissue strands (black arrows) and showing complete suppression on fat-saturated sequence (asterisk). Additional T2-hypointense intramural nodules are noted along the periphery of the lesion (yellow arrows) (c, d). STIR: short tau inversion recovery, MRI: magnetic resonance imaging

MRI (Figure 3) demonstrated a well-defined lesion arising from the uterine fundus, appearing markedly hyperintense on both T1- and T2-weighted images with complete suppression on fat-suppressed/short tau inversion recovery (STIR) sequences, confirming the presence of macroscopic fat. A few thin internal hypointense soft tissue strands were noted within the lesion, corresponding to smooth muscle components. Multiple additional T1/T2 hypointense intramural fibroids were also noted peripherally around the main lesion. No imaging features suspicious for aggressive behavior were identified on the available sequences.

The presence of subtle internal soft tissue strands and additional peripheral intramural fibroids favored the diagnosis of fat-predominant lipoleiomyoma over a pure uterine lipoma. Based on these imaging features, a diagnosis of fat-predominant lipoleiomyoma was favored.

Given the large size of the lesion, progressive abdominal fullness, and completed family status, the patient subsequently underwent total abdominal hysterectomy. Histopathological evaluation revealed a well-encapsulated lesion composed predominantly of mature adipocytes with interspersed benign smooth muscle bundles without atypia, necrosis, or significant mitotic activity, confirming the diagnosis of fat-predominant lipoleiomyoma (histopathology images not available).

## Discussion

Lipoleiomyoma is a rare benign uterine tumor composed of variable proportions of smooth muscle cells and mature adipocytes. It is considered a lipomatous variant of leiomyoma and typically occurs in perimenopausal and postmenopausal women [1,2]. Fat-predominant variants are even less frequently reported [1,3]. Although many lesions are incidental, larger masses may produce abdominal distension, pelvic pain, or pressure effects on adjacent structures. Their clinical presentation may overlap with other uterine pathologies, making imaging crucial for accurate diagnosis.

Ultrasound typically reveals a hyperechoic uterine mass with minimal vascularity. CT helps confirm the presence of fat attenuation within the lesion. MRI is particularly useful for tissue characterization, showing hyperintensity on T1- and T2-weighted images with signal suppression on fat-saturated sequences, confirming the fatty nature of the lesion. The presence of internal soft tissue elements assists in distinguishing lipoleiomyoma from pure lipoma, which typically lacks appreciable non-fatty soft tissue components [2,4,5]. In addition, the coexistence of conventional uterine leiomyomas or peripheral fibroid nodules may further support the diagnosis of lipoleiomyoma, reflecting its origin as a lipomatous variant of leiomyoma rather than a true pure lipomatous neoplasm.

Although both are benign entities, and large lesions may warrant surgical removal regardless of histological subtype, distinguishing lipoleiomyoma from pure uterine lipoma remains important for accurate radiologic characterization. Pure uterine lipomas are exceptionally rare, making accurate radiologic distinction important.

Additional differential considerations for fat-containing pelvic lesions include ovarian dermoid, pelvic lipomatosis, and atypical lipomatous tumor/well-differentiated liposarcoma. Ovarian dermoids commonly contain fat-fluid levels, calcifications, or Rokitansky nodules and are typically adnexal in origin. Pelvic lipomatosis typically demonstrates diffuse non-encapsulated pelvic fat proliferation rather than a well-circumscribed uterine mass. Well-differentiated liposarcomas may demonstrate thick septations, nodular soft tissue components, or invasive features, which were absent in the present case. Recognition of the lesion origin, internal architecture, and associated uterine fibroids assists in narrowing the differential diagnosis.

A brief comparison with previously reported cases is summarized in Table 1, highlighting the clinical presentation, imaging features, differential diagnoses, and management of fat-predominant uterine lipoleiomyoma.

**Table 1:** Comparison of previously reported cases of fat-predominant uterine lipoleiomyoma and the present case MRI: magnetic resonance imaging, CT: computed tomography

Study	Age	Clinical presentation	Imaging findings	Differential diagnosis	Management
Wilke et al. [1]	58 years	Incidental abdominal pain	Fat-containing uterine lesion on CT and ultrasound with claw sign	Ovarian dermoid cyst	Hysterectomy
Oh et al. [2]	45-70 years	Mostly nonspecific symptoms	Fat-containing uterine masses on CT/MRI	Pelvic mass/teratoma/sarcoma	Surgical management
Rampersad et al. [3]	Elderly female	Pelvic mass symptoms	Predominantly fatty uterine lesion with soft tissue elements	Lipoleiomyoma versus lipoma	Hysterectomy
El Hassouni et al. [4]	Middle-aged female	Pelvic pain	Fat-containing uterine mass on MRI	Dermoid cyst	Surgical excision
Present case	44 years	Gradually progressive lower abdominal fullness	Predominantly fatty intramural uterine lesion with subtle internal soft tissue strands and complete fat suppression on MRI	Pure uterine lipoma	Total abdominal hysterectomy

## Conclusions

Fat-predominant uterine lipoleiomyoma is a rare benign uterine lesion that may closely mimic other fat-containing pelvic masses on imaging. Recognition of subtle imaging features, such as internal soft tissue strands and uterine origin, can aid in accurate differentiation from entities such as pure lipoma or ovarian dermoid. Multimodality imaging plays an important role in improving diagnostic confidence and guiding appropriate management.
